# Assessing Self-Management Coping in Urinary Incontinence: Psychometric Evaluation of the UI-SMCSI in Middle-Aged Women

**DOI:** 10.1007/s00192-025-06274-z

**Published:** 2025-09-25

**Authors:** Marta G. Porto, Filipa Pimenta, Inês Queiroz-Garcia, Marta Uva, Matilde Patrone, Teresa Mascarenhas, João Marôco

**Affiliations:** 1 William James Center for Research, Ispa – Instituto Universitário, Rua Jardim do Tabaco, 34,, 1149-041 Lisbon, Portugal; 2 Ispa – Instituto Universitário, Lisbon, Portugal; 3 Department of Obstetrics and Gynecology, CHSJEPE/Faculty of Medicine, University of Porto, Porto, Portugal; 4 Intrepid Lab, ECEO – Universidade Lusófona & CETRAD, Lisbon, Portugal

**Keywords:** Urinary incontinence (UI), Coping strategies, Self-management, Psychometric validation

## Abstract

**Introduction and Hypothesis:**

Urinary incontinence (UI) significantly impairs women’s quality of life and often leads to the adoption of self-management coping strategies. This study aimed to develop and validate the UI Self-Management Coping Strategies Instrument (UI-SMCSI) to assess coping strategies in women with UI.

**Method:**

A sample of 1538 Portuguese women aged 40–65 years with self-reported UI participated in the study. A quantitative design was employed to evaluate the instrument’s factor structure, multigroup invariance, internal consistency, and validity evidence based on its internal structure and relations to other variables.

**Results:**

Confirmatory factor analysis supported a bidimensional first-order structure comprising defensive and hiding strategies, with excellent model fit indices (CFI = .989; TLI = .987; RMSEA = .072). Internal consistency was strong (α_defensive = .94; α_hiding = .80). Measurement invariance was confirmed across UI subtypes (stress, urge, and mixed). Convergent validity was supported by a moderately strong correlation (*r* = .615, *p* < .001) with the severity measures dimension of the King’s Health Questionnaire.

**Discussion:**

The UI-SMCSI is a reliable and valid instrument for assessing UI self-management coping strategies among Portuguese women with UI. It offers a practical tool for healthcare professionals to identify behavioral patterns that may hinder effective UI management. Future research should explore the scale’s sensitivity to intervention and its applicability to other demographic and cultural populations.

**Supplementary Information:**

The online version contains supplementary material available at 10.1007/s00192-025-06274-z.

## Introduction

Urinary incontinence (UI), defined by the International Continence Society as the complaint of any involuntary loss of urine, is a prevalent and often distressing condition that disproportionately affects women [1]. More than a physiological dysfunction, UI impairs psychosocial functioning, restricts daily activities, and undermines quality of life (QoL) through stigma, embarrassment, and social withdrawal [2–4].

UI is clinically classified into three major subtypes: stress UI (SUI), typically associated with urethral hypermobility or sphincter weakness; urgency UI (UUI), linked to detrusor overactivity; and mixed UI (MUI), which presents features of both [5]. Studies consistently show that MUI and UUI are associated with greater psychological distress than SUI, especially due to their unpredictability and higher perceived burden [6, 7]. According to the EPINCOT study, most women report mild (42.1%) to moderate (45.5%) symptoms, with approximately 10.5% experiencing severe UI [8].

The psychosocial implications of UI are equally significant. Recent data show that 50% of women with MUI and 40% with UUI report anxiety, while 16.8% of those with SUI experience depressive symptoms [3]. These mental health effects are often exacerbated by the emotional burden of embarrassment, fear of public accidents, and reduced social engagement [9, 10].

Despite the burden of UI, only around one in four women with symptoms seek professional medical treatment, due in part to stigma, shame, cultural beliefs, and the normalization of symptoms as part of aging [11, 12]. Consequently, the majority turn to self-management strategies to cope with their symptoms and maintain daily functionality.

These self-management strategies range from functional—such as adjusting fluid intake, planning bathroom access, or using containment products—to dysfunctional, including avoidance of physical or social activity, excessive concealment, and complete resignation. While some may maintain short-term social normalcy, these maladaptive responses often delay diagnosis, worsen symptoms, and reduce quality of life.

A notable empirical foundation was laid by Diokno et al. [13], who conducted a large-scale, population-based study in the United States identifying a wide range of coping strategies among women with UI. These included frequent voiding, fluid restriction, use of pads, concealment through clothing, and avoidance of social activities, which were categorized into three domains: hiding strategies, defensive strategies, and treatment strategies. While treatment strategies (e.g., pelvic floor exercises done according to recommendations and, preferably with validations/verification by a specialist health professional, or medication use) are considered adaptive, many hiding and defensive responses represent dysfunctional coping—behaviors that may preserve “social continence” [14], but ultimately hinder the implementation of UI adaptative mitigation behaviors, and delay help seeking and subsequent diagnosis. Despite the value of this typology, Diokno’s framework was not developed into a validated instrument, and it included medical strategies that may not reflect the self-management strategies women employ in their daily lives.

While existing instruments tend to assess the impact of UI on QoL, only a few address the coping strategies women use to manage UI symptoms directly and in the context of their daily lives. Among the limited tools available, only two are condition-specific: Aydin Avci et al. [15] developed the *Frequency of the Use of Non-Medication Coping Strategies for Urinary Incontinence Questionnaire*, targeting older adults and incorporating 16 items across bodily, psychological, social, and material domains, Xu et al. [16], building on Wu et al. [17], employed the *Incontinence Coping Style Questionnaire* (I-CSQ) to evaluate three coping types—avoidant, palliative, and instrumental—but it remains available only in Chinese. Other instruments such as the *Measure of Adaptations for Pelvic Symptoms* (MAPS, Wren et al. [18]) and the *Adaptive Behavior Index* (ABI, [19] are broader in scope, assessing coping in pelvic floor disorders more generally, and do not isolate behaviors specific to UI.

Given the scarcity of validated, culturally and contextually appropriate instruments that assess nonmedical coping strategies for UI—particularly in middle-aged women—this study aims to develop and validate the UI Self-Management Coping Strategies Instrument (UI-SMCSI). Grounded in Diokno et al.’s [13] empirical typology but refined to capture exclusively self-management behaviors, the UI-SMCSI is designed as a patient-reported outcome measure (PROM) to systematically assess UI self-management coping strategies, which might be dysfunctional. In the absence of structured strategies aimed at mitigating UI—such as pelvic floor muscle training or engagement with professional medical care—this instrument serves to enhance the systematic assessment of self-management behaviors. It is designed to support the development of individualized clinical guidance and tailored symptom management strategies. Owing to its brevity and targeted scope, the instrument offers practical utility for both clinical and research applications, facilitating efficient data collection without compromising the specificity required for meaningful interpretation.

## Method

### Design

For this study, an observational, cross-sectional, descriptive, and correlational design was used.

### Participants

A non-probabilistic sample of 1606 women who self-reported occasional or frequent urine loss (*M*_age_ = 50.19, *SD*_age_ = 6.58) were recruited. The inclusion criteria for this study were: (1) Female sex; (2) Aged between 40 and 65 years; (3) Experiencing involuntary urine loss either during intra-abdominal pressure increases and/or when feeling an uncontrollable urge to urinate, occasionally or frequently; and (4) Having access to the internet. Exclusion criteria included: (1) Being pregnant or less than 6 months postpartum; (2) Having undergone UI-related surgery; (3) Having an oncological disease; (4) Having neurological disabilities; and (5) Having experienced pelvic organ prolapse.

The final analytical sample consisted of 1538 women, as 56 participants were excluded due to previous UI-related surgeries and 12 women declined to provide informed consent.

### Measures

The Questionnaire for Urinary Incontinence Diagnosis (QUID; [20], specifically designed for identifying UI in women, was administered to assess the different UI types. The diagnostic threshold was set at ≥ 4 for stress UI and ≥ 6 for urge UI. Consequently, mixed UI classification required meeting both criteria simultaneously. This scale consists of six items: three corresponding to stress incontinence symptoms and three related to urge incontinence symptoms. Each item is rated on a Likert-scale, ranging from 0 (“none of the time”) to 5 (“all of the time”). The total score for each dimension ranges from 0 to 15. The QUID demonstrated moderate to good internal consistency, with α =.64 for stress UI and α =.87 for urge UI [20].

The International Consultation on Incontinence Questionnaire-Urinary Incontinence Short Form (ICIQ-UI SF; [21] was used to assess the frequency and amount of urinary leakage, as well as its overall impact on daily life. This questionnaire consists of three scored questions addressing these aspects, with sample items such as: “How often do you leak urine?” or “How much urine do you usually leak (whether you wear protection or not)?”. Responses are summed to yield an overall score ranging from 0 to 21, where higher scores indicate more severe UI symptoms. Additionally, the ICIQ-UI SF includes a fourth, unscored question that identifies the circumstances in which leakage occurs. The total score on the ICIQ-UI Short Form can be interpreted categorically to reflect symptom severity: scores of 1–5 indicate slight incontinence, 6–12 moderate, 13–18 severe, and 19–21 very severe. The instrument has demonstrated excellent internal consistency reliability (α = 0.95, [21].

The King’s Health Questionnaire (KHQ; [22] was used to assess the impact of UI on health-related quality of life, being a multidimensional patient-reported outcome measure (PROM) widely used in both clinical and research settings. Developed to capture the psychosocial, physical, and relational consequences of UI, the KHQ has demonstrated robust psychometric properties across diverse populations and has been validated for use in Portuguese [23]. The KHQ consists of 21 items divided across three parts and nine domains, each scored on a scale of 0 to 100, where higher scores indicate greater perceived impairment: Part I includes two domains:

1. General Health Perception, assessed via a five-point scale from *very good* to *very poor*, and
2. Incontinence Impact, rated on a four-point scale measuring perceived burden (*not at all* to *a lot*);

Part II assesses six domains reflecting functional and psychosocial limitations, including:

(3) Role Limitations,
(4) Physical Limitations,
(5) Social Limitations,
(6) Personal Relationships,
(7) Emotions,
(8) Sleep/Energy and
(9) Severity Measures.

Each of these domains comprises two to three items answered on a four-point Likert scale (*not at all* to *all the time* or equivalent wording), allowing fine-grained analysis of the multifaceted burden of UI. Part III includes the Symptom Severity Scale, composed of ten sub-items assessing the frequency and severity of core urinary symptoms such as nocturia, urgency, stress and urge incontinence, bedwetting, intercourse incontinence, urinary infections, dysuria, and dribbling.

The UI Self-Management Coping Strategies Instrument (UI-SMCSI) was designed to measure nonmedical coping strategies employed by women with urinary incontinence (UI), based on the framework by Diokno et al. [13]. The initial 16-item scale encompassed two dimensions: *hiding coping* (concealing urine loss) and *defensive coping* (preventing leakage through restrictive or anticipatory behaviors). Items were rated on a five-point Likert scale. After confirmatory factor analysis and item refinement, 13 items were retained in the final version, with higher scores indicating greater use of potentially maladaptive strategies. An additional section assessed treatment history and care-seeking behaviors. Instrument development and validation are detailed in the “Results” section.

### Procedure

This study’s data was collected during the COVID-19 pandemic (March to October 2020) using the online survey platform Google Forms, which included the previously described measures. Owing to pandemic-related restrictions, a contingency plan was implemented as an alternative to the original hospital-based recruitment strategy. To recruit participants, invitations were distributed via social media, primarily Facebook. A concise summary of the research objectives was shared across various groups of middle-aged and menopausal women to encourage participation.

### Data Analysis

Statistical analyses for the present study were conducted in two distinct phases, aligning with the adaptation and validation process of the *Urinary Incontinence Self-Management Coping Strategies Instrument* (UI-SMCSI). Phase 1 focused on the adaptation of items from an existing instrument, ensuring content relevance and clarity for the target population. Phase 2 encompassed comprehensive psychometric evaluation.

To guide the validation process in phase 2, the study followed the *Standards for Educational and Psychological Testing* [24]. In accordance with these guidelines, two primary sources of validity evidence were examined for the UI-SMCSI:

1. **Evidence based on internal structure**, including analyses of dimensionality, internal consistency reliability, and measurement invariance; and
2. **Evidence based on relations to other variables**, assessing construct validity through correlations with theoretically relevant constructs.

Data analyses were conducted using R 4.0 (R Core Team) via RStudio, and IBM SPSS Statistics 27.0. Descriptive statistics—including mean (*M*), standard deviation (*SD*), skewness (*Sk*), and kurtosis (*Ku*)—were computed to evaluate the distributional properties of each item. Multivariate normality was tested using the *MVN* package [44]. Items were flagged for severe non-normality if |*Sk*| > 3 or |*Ku*| > 7 [25]. Sample size estimation followed the recommendations of Hair et al. [26], suggesting a minimum of 5 to 10 participants per manifest variable (i.e., between 80 and 160 participants, considering the initial 16 items), thereby ensuring adequate statistical power.

Given the strong theoretical foundation in the construction of the items and its factors, the present study employed confirmatory factor analysis (CFA) as the primary analytic strategy to assess the internal structure of the UI-SMCSI. The use of CFA is well-supported by existing literature and psychometric standards, particularly when the relationships between latent constructs and observed indicators are theoretically grounded. As such, CFA enables a direct test of model fit, providing a rigorous assessment of whether the hypothesized two-factor structure—comprising *hiding* and *defensive* coping dimensions—accurately reflects the underlying conceptual framework [27].

The CFA was conducted using the diagonally weighted least squares (DWLS) estimator, appropriate for ordinal data, through the Lavaan package (v.0.6-14) in R [28, 29]. Model refinement involved correlating residuals based on both theoretical rationale and modification indices exceeding 11 (*p* <.001). Goodness-of-fit was evaluated using multiple indices: Comparative Fit Index (CFI) ≥.90, Tucker–Lewis Index (TLI) ≥.90, root mean square error of approximation (RMSEA) ≤.08, and standardized root mean square residual (SRMR) ≤.08 [25, 30]. Internal structure validity evidence was also evaluated using the criterion that the average variance extracted (AVE) exceeded.50, in line with Fornell and Larcker [31] guidelines. Additionally, it was confirmed that the AVE for each factor was greater than the squared correlations between factors.

Internal consistency reliability was assessed using ordinal α—calculated from the polychoric correlation matrix—and McDonald’s ω. Coefficients ≥.70 were considered acceptable [25]. The package semTools was used to estimate the reliability indices.

To test measurement invariance, two multigroup confirmatory factor analyses (MGCFA) were conducted. First, the full sample was randomly split into two subsamples (50% each) using SPSS, and a sequence of nested models—configural, metric, scalar, and strict—was evaluated. Second, a separate MGCFA tested invariance across UI subtypes (stress, urge, and mixed). Both analyses used the robust maximum likelihood estimator (MLR) in Lavaan as this estimator performs well with ordinal data nonseverely biased [29]. Invariance was assumed when changes in Comparative Fit Index (ΔCFI) were <.01 [32] and changes in RMSEA (ΔRMSEA) were <.02 [33].

To examine validity evidence based on relations to other variables, convergent validity was assessed through Pearson correlation analyses. Specifically, we tested the association between the total score of the UI Self-Management Coping Strategies Instrument (UI-SMCSI) and selected items from the King’s Health Questionnaire (KHQ) Severity Measures dimension—namely, item 17 (“Do you wear pads to keep dry?”), item 18 (“Do you have to be careful about how much fluid you drink?”), and item 19 (“Do you change your underclothes because they get wet?”). These items were chosen due to their conceptual alignment with the behavioral content captured in the UI-SMCSI, particularly regarding nonmedical self-management behaviors. Given the scarcity of validated instruments that directly assess UI self-management coping strategies, these items offer a meaningful point of comparison.

## Results

### Brief Sample Clinical Characterization

Among the 1538 women with UI included in the study, the majority had experienced one or two vaginal deliveries (61.2%), and more than half (54.6%) reported a history of perineal laceration. Most participants were in the peri- or postmenopausal stage (77.3%). Overweight and obesity were common, affecting 56.5% of the sample. Physical illnesses were reported by 34.4%, and 14.7% had a diagnosed mental health condition, most frequently depression (8.8%) and anxiety (4.7%). Stress UI was the most prevalent subtype (36.2%), followed by urgency (21.7%) and mixed UI (16.1%). Regarding severity, based on the ICIQ-UI SF, 38.5% of participants were classified as having slight UI, 40.6% moderate, 15.1% severe, and 5.8% very severe.

Regarding *medical management strategies*, although 41% of women reported seeking professional support for urinary incontinence, only 29.1% received any form of treatment. Furthermore, just 24% reported engaging in pelvic floor muscle training, and only 4.6% were using prescribed medication (Tables 1, 2, and 3).

**Table 1:** Sociodemographic characteristics of participants

Characteristics	Women with UI (*n* = 1538)	
	*N*	%
*Age range (in years)*		
40−44	353	23.3
45−49	392	25.9
50−54	368	24.3
55−59	266	15.9
60−65	159	10.5
*Nationality*		
Portuguese	1482	96.3
Other	56	3.7
*Level of education*		
4th grade	6	0.4
9th grade	118	7.8
12 h grade	447	29.5
Bachelor’s degree	573	37.9
*Postgraduate degree*	358	22.0
Doctorate	36	2.4
*Professional status*		
Active	1245	80.6
Inactive	293	19.4
*Sexual-affective relationship*		
Yes	1222	79.1
No	316	20.9
*Number of biological children*		
0	152	10.0
1	462	28.9
2	695	45.9
3	185	12.2
> 4	44	2.9

*UI* Urinary incontinence

**Table 2:** Clinical characteristics and lifestyle habits of participants

Characteristics	Women with UI (*n* = 1538)		
	N	%	
*Number of vaginal deliveries*			
0	425	28.1	
1	442	27.6	
2	509	33.6	
3	131	8.7	
4	31	2.0	
*Number of caesarean deliveries*			
0	1062	70.2	
1	325	19.8	
2	121	8.0	
3	28	1.9	
4	2	0.1	
*Perineal laceration*			
Yes	851	54.6	
No	687	45.4	
*Physical illness diagnosis*^a^			
No	993	65.6	
Yes	545	34.4	
Asthma	45	2.9	
Hypothyroidism	51	3.3	
Diabetes	35	2.2	
Hypertension	72	4.7	
*Mental illness diagnosis*^a^			
No	1290	85.3	
Yes	248	14.7	
Depressive disorder	133	8.8	
Anxiety disorder	96	4.7	
Obsessive disorder	4	0.3	
Psychotic disorder	5	0.3	
*Menopausal status*			
Premenopause	349	22.7	
Perimenopause	559	36.3	
Postmenopause	630	41.0	
*Medical help to manage menopausal symptoms*			
Yes	664	43.9	
No	874	56.1	
*Coffee intake*			
Yes	1263	83.5	
No	275	16.5	
*Hot/cold beverages intake*			
Yes	1286	85.0	
No	252	15.0	
*High impact physical activity*			
Yes	207		13.7
No	1331		86.3
*BMI status*			
Underweight (BMI < 18.5 kg/m^2^)	32		2.1
Normal weight (BMI 18.5–24.9 kg/m^2^)	651		41.4
Overweight (BMI 25.9–29.9 kg/m^2^)	552		36.5
Obesity class I (BMI 30–34.9 kg/m^2^)	226		14.9
Obesity class II (BMI 35–39.9 kg/m^2^)	60		4.0
Obesity class III (BMI > 40 kg/m^2^)	17		1.1
*UI types*			
Stress	558		36.2
Urgency	334		21.7
Mixed	243		16.1
*UI severity*			
Mild to moderate	1200		78.0
Moderate to severe	338		22.0
*ICIQ-UI SF levels*			
Slight [1–5]	592		38.5
Moderate [6–12]	624		40.6
Severe [13–18]	233		15.1
Very severe [19–21]	89		5.8
*Medical help for UI*			
Yes	620		41.0
No	918		59.0
*Treatment for UI*			
Yes	448		29.1
No	1090		70.9
*Prescribed UI medication*			
Yes	70		4.6
No	1468		95.4
*Pelvic floor muscle training*			
Yes	369		24
No	1169		76

*UI* Urinary incontinence. ^a^ Most prevalent self-reported diagnosis

**Table 3:** Severity and UI types per menopause status

Menopause status	Severity		UI types			
	Mild to moderate	Moderate to severe	Stress	Urgency	Mixed	Below UI stress/urgency scores
	*n* (%)	*n* (%)	*n* (%)	*n* (%)	*n* (%)	*n* (%)
Premenopausal	258 (21.5)	91 (26.9)	87 (15.6)	39 (11.7)	30 (12.3)	193 (47.9)
Perimenopausal	424 (35.3)	135 (39.9)	242 (43.4)	144 (43.1)	103 (42.4)	65 (16.1)
Posmenopausal	518 (43.2)	112 (33.2)	229 (41.0)	151 (45.2)	110 (45.3)	145 (36.0)

*UI* Urinary incontinence

*Regarding self-management coping strategies*, the most frequently endorsed behaviors included the use of feminine hygiene pads (hiding dimension; *M* = 1.96, *Md* = 2.00), immediately locating the bathroom upon entering an unfamiliar environment (defensive; *M* = 1.18, *Md* = 1.00), reducing fluid intake (defensive; *M* = 1.15, *Md* = 1.00), and frequent voiding even in the absence of urgency to maintain an empty bladder (defensive; *M* = 1.13, *Md* = 1.00). These findings suggest a preference for practical, anticipatory strategies aimed at managing symptoms and minimizing the risk of leakage. In contrast, strategies indicative of avoidance or concealment were less commonly reported. These included limiting social outings (defensive; *M* = 0.48, *Md* = 0.00), avoiding unfamiliar places (defensive; *M* = 0.74, *Md* = 0.00), and wearing dark clothing to mask potential stains (hiding; *M* = 0.58, *Md* = 0.00) (see Table 4 and Appendix C). The sociodemographic characteristics of the participants are presented in Table 1.

**Table 4:** Descriptive statistics of the UI-SMCSI items

	Item	*M*	*SD*	Min	Max	*Sk*	*Ku*
**Defensive coping**	Item 1	1.13	1.03	0	4	.63	−.30
	Item 2	1.17	1.06	0	4	.15	.23
	Item 3	1.15	1.11	0	4	.65	−.48
	Item 4	.74	1.02	0	4	1.42	1.41
	Item 5	.61	.96	0	4	1.69	2.33
	Item 6	.84	1.10	0	4	1.23	.60
	Item 7	.72	1.07	0	4	1.49	1.37
	Item 8	.48	.85	0	4	2.02	3.97
	Item 9	.55	.93	0	4	1.87	3.04
**Hiding coping**	Item 10	.10	.46	0	4	5.54	34.95
	Item 11	1.96	1.47	0	4	.10	−1.34
	Item 12	.73	1.00	0	4	1.33	1.13
	Item 13	.58	1.03	0	4	1.80	2.33
	Item 14	.37	.85	0	4	2.49	5.71
	Item 15	.15	.61	0	4	4.67	22.72
	Item 16	.21	.64	0	4	3.59	13.83

Additionally, information on clinical details, lifestyle-related factors, and UI (Table 2) was gathered.

The guidelines outlined in the Stages of Reproductive Aging Workshop (STRAW; [34] were used to determine participants’ menopausal stages. On the basis of their self-reports, participants were classified into three categories: (1) premenopausal, no changes in menstrual cycle regularity, (2) perimenopausal, characterized by variable cycle lengths (deviating by more than 7 days from their typical cycle) or the absence of two or more cycles, resulting in an amenorrhea period of more than 60 days; and (3) postmenopausal, defined as having experienced at least 12 consecutive months of amenorrhea (Table 3 presents the classification details).

### Phase 1: Instrument Development

Conceptually grounded in the framework proposed by Diokno et al. [13], the initial version of the UI-SMCSI consisted of 16 items (Appendix A), organized into two theoretically distinct dimensions:

1. Hiding Coping (7 items): This dimension reflects strategies aimed at concealing visible signs of urine leakage, particularly in social or public settings. These behaviors are often oriented toward minimizing embarrassment or avoiding stigma. Illustrative items include: *“Wearing dark clothes to hide urine stains,” “Using feminine hygiene pads,”* and *“Carrying extra underwear or clothing.”*
2. Defensive Coping (9 items): This dimension captures anticipatory or restrictive behaviors intended to prevent or control urine loss. These strategies often involve modifications to daily routines or social engagement to reduce the risk of leakage. Representative items include: *“Limiting fluid intake,” “Avoiding social activities,”* and *“Voiding frequently to stay dry.”*

Together, these dimensions reflect distinct yet interrelated coping responses that women may adopt in the absence of or alongside formal medical intervention for urinary incontinence.

Each item was rated on a five-point Likert scale, ranging from 1 (“never”) to 5 (“always”), with total scores in the initial version ranging from 16 to 80. Following confirmatory factor analysis and item refinement—described in detail in “Phase 2: Instrument Validation” (see “Results” section)—the final version retained 13 items, resulting in a revised total score range of 13 to 65. Higher scores indicate a greater reliance on potentially maladaptive self-management strategies.

Moreover, to provide a more comprehensive understanding of participants’ UI self-management, a brief questionnaire was included to assess treatment history and clinically oriented behaviors. This section captured key contextual information, including current use of UI-related medication (yes/no; if yes, specify); engagement in pelvic floor muscle training (e.g., Kegel exercises); history of surgical interventions for UI; and whether the participant had discussed their condition with a healthcare professional. When applicable, participants were asked to report any recommendations they received from healthcare providers. These items are critical for situating self-management coping strategies within the broader context of formal care-seeking, clinical guidance, and treatment engagement among women with UI.

### Phase 2: Instrument Validation

#### Validity Evidence Based on the Internal Structure

*Dimensionality*.

##### Items’ Distributional Properties

After analyzing the descriptive statistics of all items on the UI Self-Management Coping Strategies Instrument (Table 4), it was found that items 10, 15, and 16 presented severe violations of normality. Therefore, its exclusion was considered. However, these three items could be used in future studies with clinical samples. Apart from these three items, no other severe violations of normality were found as all the items exhibited adequate absolute values of skewness (1.04 ≤ |*Sk*| ≤ 2.5) and kurtosis (.29 ≤ |*Ku*| ≤ 3.94).

##### Factor Related Validity Evidence

**Fig. 1:**
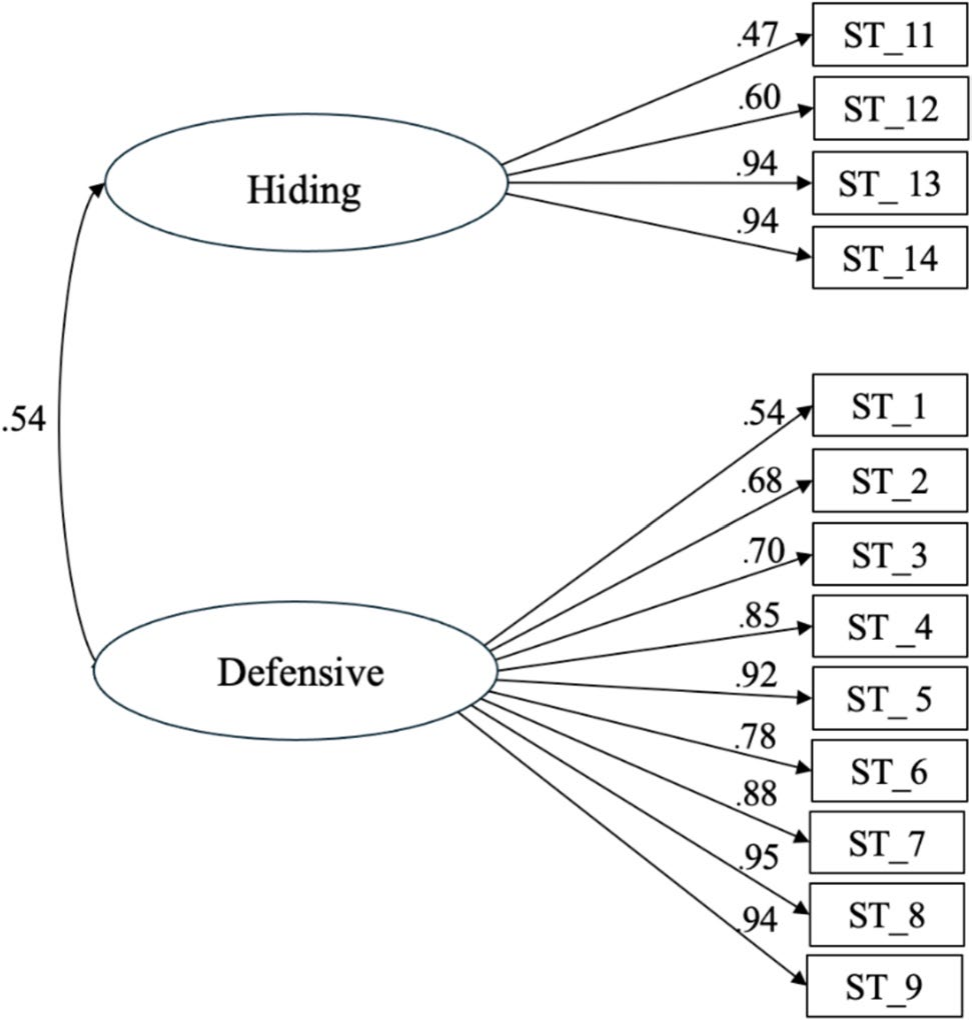
First-order latent factor structure of the reduced 13-item version of the UI-SMCSI in a sample of Portuguese women (*n* = 1538). *Note*. Numeric values represent standardized factor loadings for each item

After excluding these three items, all remaining items demonstrated adequate and significant standardized factor loadings (.47 ≤ λ ≤.95) and individual reliability. The UI-SMCSI showed strong validity evidence based on internal structure, as indicated by the excellent goodness-of-fit indices (CFI =.989; TLI =.987; RMSEA =.072; SRMR =.039; see Fig. 1).

The average variance extracted (AVE) for the defensive (.69) and hiding (.54) dimensions exceeded their shared variance (*r*^*2*^ =.402), also providing validity evidence based on internal structure and confirming that the two dimensions represent structurally distinct constructs. As such, the second-order factor model was rejected in favor of a bidimensional first-order solution.

##### Internal Consistency Reliability

Internal consistency estimates were used to assess the reliability of the scores. The defensive (α_ordinal_ =.94, ω =.92) and hiding (α_ordinal_ =.80, ω =.83) coping dimensions demonstrated excellent and good internal consistency, respectively. These results indicate that the item scores are consistently clustered around the latent variable for UI strategies.

##### Measurement Invariance of the UI-SMCSI

The measurement invariance of the UI-SMCSI was tested using multigroup confirmatory factor analysis (MGCFA). For this purpose, the total sample was randomly divided into two equal subsamples (50–50%) using SPSS randomization procedures (see Table 5). A series of hierarchically nested models were estimated to assess increasingly restrictive levels of invariance. The results supported configural, metric, scalar, and strict invariance across the two subsamples. These findings indicate that the UI-SMCSI demonstrates a stable factor structure across independent groups, providing additional validity evidence based on internal structure.

**Table 5:** Measurement invariance of the UI-SMCSI

Model	Df	χ^2^	Δχ^2^	ΔDf	*P*[Δχ^2^>]	CFI	RMSEA	ΔCFI	ΔRMSEA
Configural	128	422.777	—	—	—	.997	.055	.000	.000
Metric	139	462.028	15.245	11	.172	.997	0.055	.000	.000
Scalar	176	489.726	52.175	37	.050	.997	0.049	.000	-.007
Strict	178	528.925	8.351	2	.015	.996	0.051	.000	.003

*Df* degrees of freedom; χ^2^ = chi-square value; Δχ^2^ = difference in chi-square from the previous model; ΔDf = difference in degrees of freedom from the previous model; *P*[Δχ^2^>] = *p* value for the chi-square difference test

##### Structural Invariance of the UI-SMCSI Across UI Types

To assess whether the original one-factor model holds across different UI types and severity levels (Table 6), a series of nested models with indications of equivalence were required. This analysis accounts for the ordinal nature of the scales. Therefore, a structural invariance analysis was conducted across UI types and severity.

**Table 6:** Multigroup invariance of the one-factor model of the UI-SMCSI across UI types

Model	Df	χ^2^	Δχ^2^	ΔDf	*P*[Δχ^2^>]	CFI	RMSEA	ΔCFI	ΔRMSEA
Configural	212	377.308	—	—	—	.998	.045	.000	.000
Metric	242	552.061	76.466	30	.000	.996	.058	-.002	.013
Scalar	344	819.500	380.580	102	.000	.994	.061	-.002	.002
Strict	350	2354.883	391.625	6	.000	.975	.123	-.019	.063

*Df* degrees of freedom; χ^2^ = chi-square value; Δχ^2^ = difference in chi-square from the previous model; ΔDf = difference in degrees of freedom from the previous model; *P*[Δχ^2^>] = *p* value for the chi-square difference test

### Validity Evidence Based on Relations to Other Variables

#### Convergent Validity Evidence

A Pearson correlation analysis was conducted between the total score of the UI-SMCSI and the total score of the 8th dimension (“Do you do any of the following?”) from the KHQ. The analysis revealed a strong correlation between the two total scores (*r* =.615, *p* <.001). This finding provides convergent validity evidence for the instrument, demonstrating a strong association with scores from other tests that assess the same construct.

## Discussion

The current study aimed to validate the psychometric properties of the UI Strategies Instrument for Portuguese women (UI-SMCSI; Appendix B), specifically evaluating its reliability, validity, and measurement invariance. Furthermore, we sought to analyze the multigroup invariance of the proposed one-factor model across different types of UI, establishing a robust basis for assessing dysfunctional coping strategies associated with UI.

In line with our objectives, the results confirmed a bidimensional first-order structure of the 13-item version of the UI-SMCSI (AVE_Defensive_ =.69 and AVE_Hiding_ =.54; > *r*^*2*^ =.402), yielding excellent goodness-of-fit indices (CFI =.989; TLI =.987; RMSEA =.072; SRMR =.039). Internal consistency reliability estimates (defensive α =.94; ω =.92; hiding α =.80; ω =.83) further reinforced the scale’s robustness. Convergent validity was established through a strong correlation with the King’s Health Questionnaire’s (KHQ) Severity Measures Dimension (*r* =.615, *p* <.001), a gold-standard instrument with grade A recommendations from the International Consultation on Incontinence. This supports the instrument’s ability to assess self-management coping strategies effectively.

These findings provide strong validity evidence based on internal structure, confirming that the UI-SMCSI functions consistently across independent samples and can be confidently applied in diverse clinical or research settings.

These findings align with previous validation efforts of UI-related tools, such as the International Consultation on Incontinence Questionnaire-Urinary Incontinence short form (ICIQ-UI SF) and the Measure of Adaptations for Pelvic Symptoms (MAPS), which have also demonstrated high reliability and validity across diverse populations [20, 46]. Notably, our results revealed that all but three items exhibited adequate standardized factor loadings (.47 ≤ λ ≤.95) and individual reliability, validating their relevance in capturing dysfunctional coping strategies. Items 10, 15, and 16 were excluded due to violations of normality; however, it is recommended that the scale be used in its original version, as developed by Diokno et al. [13], to preserve conceptual completeness.

Our findings also showed that regarding the self-management coping strategies most used by our sample, the results indicate that women predominantly adopted defensive strategies, such as frequent voiding, reducing fluid intake, and locating bathrooms in unfamiliar settings. These behaviors reflect a preventive and control-oriented approach aimed at minimizing the risk of leakage, rather than avoiding situations or concealing symptoms. Although hiding strategies were generally less endorsed, the most frequently used individual item overall—the use of feminine hygiene pads—belongs to the hiding dimension, suggesting that while concealment is not the dominant coping style, practical protection remains a widely adopted and accessible method. This overall pattern may be influenced by factors such as symptom severity, perceived control, or sociocultural norms [35], 45], highlighting a preference for active self-management over social restriction or concealment. These findings are consistent with prior research emphasizing the prevalence of such strategies among women with UI [13, 36]. Moreover, the invariance of the scale across UI types underscores its versatility and suitability for application in diverse clinical contexts.

In relation to the self-management coping strategies most frequently reported in our sample, findings suggest that women primarily adopted defensive strategies, such as frequent voiding, reducing fluid intake, and identifying bathroom locations in unfamiliar environments. These behaviors reflect a preventive, control-oriented approach aimed at minimizing the risk of urinary leakage, rather than avoiding social situations or concealing symptoms. Although hiding strategies were generally less prevalent, the most frequently endorsed individual item—the use of feminine hygiene pads—was categorized within the hiding dimension. This indicates that, despite not being the dominant coping style, practical concealment through protective products remains a commonly accepted and accessible method. This pattern may be shaped by factors such as symptom severity, perceived control over symptoms, and sociocultural attitudes toward urinary incontinence, suggesting a broader preference for active self-management rather than avoidance or withdrawal.

Regarding its strengths, the UI-SMCSI offers significant clinical value as a tool for healthcare professionals, enabling the identification of coping strategies that may interfere with effective management of urinary incontinence (UI). It addresses a critical gap in the literature by providing a structured assessment of how women manage UI symptoms in the context of their everyday lives—something that has been largely underrepresented in existing measures. Its bidimensional structure—capturing both symptom-hiding behaviors and protective or defensive strategies to prevent leakage—allows for more nuanced clinical insights. This differentiation supports the development of tailored, patient-centered interventions that can more effectively address individual coping patterns. Ultimately, such personalized approaches may enhance treatment adherence and lead to improved quality of life for women living with UI.

Despite its strengths, this study has several limitations. First, although the questionnaire included supplementary items addressing treatment history, such as current medication use, engagement in pelvic floor muscle training (e.g., Kegel exercises), history of surgical intervention, and previous consultations with a healthcare provider, it did not assess critical dimensions of treatment adherence [37]. Specifically, future research should evaluate whether pelvic floor muscle exercises are performed under professional supervision, practiced with consistency (e.g., frequency per day or per week), and sustained over a clinically relevant duration (e.g., at least 3 months), as these factors are known to significantly influence therapeutic outcomes [38, 39]. Structured adherence questions (e.g., “How many days last week did you follow the behavioral advice?” or “How often did you perform the recommended exercises yesterday?”) would provide a more accurate understanding of treatment implementation.

Additionally, it is crucial to contextualize self-management strategies in terms of their adaptive or maladaptive functionality. These behaviors—such as fluid restriction, toilet mapping, or strategic use of containment products—are not inherently dysfunctional. For example, they may serve as effective symptom-management tools in contexts of limited healthcare access or while a woman is undergoing treatment. However, these same strategies may become maladaptive when used as standalone substitutes for medical consultation, particularly if they contribute to delayed diagnosis or hinder engagement with evidence-based interventions. Recognizing this nuance is essential for accurately interpreting coping strategies and for informing the development of interventions that foster adaptive self-regulation, without inadvertently reinforcing treatment avoidance or dysfunctional defensive and concealment-based coping styles [40, 41].

Future research should further explore this contextual variability, distinguishing between coping strategies that are functional and appropriate within constrained or transitional circumstances, and those that may ultimately undermine help-seeking behavior and contribute to poorer health outcomes.

Moreover, the use of a non-probabilistic sampling method limits the generalizability of the findings. Longitudinal studies are warranted to assess the scale’s responsiveness to clinical interventions and its utility in tracking treatment outcomes. Given that participants were primarily recruited via social media platforms, particularly Facebook, it is likely that our sample overrepresents women who are digitally literate and have regular internet access. This introduces potential selection bias, as these individuals may differ systematically from those who are not active on social media or lack digital resources. For instance, they may have higher levels of education, income, or health literacy, which could influence both their engagement with the intervention and the outcomes. Therefore, the generalizability of our findings to women experiencing UI—particularly those from lower socioeconomic backgrounds or with limited digital access—may be restricted. Future studies should incorporate more diverse recruitment strategies to enhance representativeness.

Another important consideration is the potential influence of neurodevelopmental conditions or forms of neurodivergence that, while not classified as severe neurological disabilities, may still impact cognitive processing, item interpretation, or coping styles. These aspects were not explicitly addressed in the current validation. Future versions of the instrument and study designs should consider including self-reported items to identify participants with confirmed or self-identified neurodivergent conditions (e.g., ADHD, autism spectrum traits). This would enable subgroup analyses to assess whether the instrument maintains its psychometric properties across diverse cognitive profiles or requires semantic adaptation to ensure validity and accessibility.

Additionally, the cross-sectional design limits the ability to assess the instrument’s test–retest reliability and its responsiveness to change over time—both crucial aspects for evaluating intervention outcomes. While the sample size was large (N = 1538), the reliance on self-reported data may introduce recall or reporting bias, particularly in the assessment of coping behaviors. Further research should explore not only the strategies themselves but also the underlying barriers that lead women to adopt them, with efforts to develop and validate additional assessment tools tailored to the Portuguese population. Incorporating objective measures, such as bladder diaries, could enrich the validity of self-reported data. Moreover, examining contextual variables—such as access to healthcare services and social support networks [42], along with beliefs [43]—may yield deeper insights into the mechanisms driving dysfunctional coping and inform more targeted intervention strategies.

In conclusion, the UI Self-Management Coping Strategies Instrument is a reliable and valid tool for assessing coping strategies in Portuguese women with UI. Its strong psychometric properties and clinical relevance contribute to a deeper understanding of UI-related strategies, supporting improved interventions and patient outcomes. Continued research will further reinforce its role in both clinical and research contexts, ultimately enhancing the management of UI.

## Supplementary Information

Below is the link to the electronic supplementary material.

Supplementary file1 (DOCX 37 KB)

## Data Availability

The data supporting this study’s findings are available from the corresponding author (MP) upon reasonable request, without undue reservation.
