# Supplementary material for: Assessing Self-Management Coping in Urinary Incontinence: Psychometric Evaluation of the UI-SMCSI in Middle-Aged Women
Source: Int Urogynecol J. 2025 Sep 25;37(2):385–96. doi: 10.1007/s00192-025-06274-z (PMC12995998; doi:10.1007/s00192-025-06274-z)
Supplement: Supplementary file 1 — Supplementary file1 (DOCX 37 KB) [file 192_2025_6274_MOESM1_ESM.docx]

**APPENDICES**

**Appendix A – Original version of the UI Self-Management Coping Strategies Instrument**

Please tick how often you have used each of the strategies indicated to manage urine loss.

|  | **Items** | **Never (0)** | **Rarely (1)** | **Sometimes (2)** | **Often**  **(3)** | **Everyday/Always (4)** |
| --- | --- | --- | --- | --- | --- | --- |
| Defensive | **1.** Going to the toilet often, even if you don't feel like it, in order to keep your bladder empty. |  |  |  |  |  |
|  | **2.** Immediately look for the toilet when you arrive in an unfamiliar place. |  |  |  |  |  |
|  | **3.** Reduce your fluid intake. |  |  |  |  |  |
|  | **4.** Avoid going to places where you don't know the location of the toilets. |  |  |  |  |  |
|  | **5.** Staying at home longer to avoid uncomfortable situations. |  |  |  |  |  |
|  | **6.** Limit physical activity. |  |  |  |  |  |
|  | **7.** Before travelling to an unfamiliar place, try to find out where the toilets are located. |  |  |  |  |  |
|  | **8.** Limit social outings. |  |  |  |  |  |
|  | **9.** Limit activities, such as travelling, that require you to be away from the toilet for a long period of time. |  |  |  |  |  |
| Hiding | **10.** Wearing nappy pants (*) |  |  |  |  |  |
|  | **11.** Use feminine (hygienic) pads. |  |  |  |  |  |
|  | **12.** Use other absorbent materials (such as toilet paper, tissues or paper towels). |  |  |  |  |  |
|  | **13.** Wear dark colours or clothes that hide the stains. |  |  |  |  |  |
|  | **14.** Wear long skirts and coats to cover the stains. |  |  |  |  |  |
|  | **15.** Use adult nappies (*) |  |  |  |  |  |
|  | **16.** Use too much perfume or cologne to disguise the smell of urine. (*) |  |  |  |  |  |
| *Note*. (*) Item deleted. | | | | | | |

**Additional Section:**

**Urinary Incontinence – Treatment and Management Questionnaire**

**1.** Do you take any medication for urinary incontinence?
 Yes ___ No ___ If yes, which one? __________

**2.** Are you currently performing pelvic floor strengthening exercises (e.g., Kegel exercises) for urinary incontinence?
 Yes ___ No ___

**3.** Have you undergone surgery to treat urinary incontinence?
 Yes ___ No ___

**4.** Have you ever talked to your doctor about your urinary incontinence?
 Yes ___ No ___

**4.1.** If yes, what was the recommendation? _________________________

**Appendix B – Portuguese version of the UI Self-Management Coping Strategies Instrument**

Por favor, assinale a frequência com que utilizou cada uma das estratégias indicadas para gerir perdas de urina.

|  | Nunca | Raramente | Algumas vezes | Muitas vezes | Todos os dias/Sempre |
| --- | --- | --- | --- | --- | --- |
| **1.** Ir muitas vezes à casa de banho, mesmo sem vontade, de forma a manter a bexiga vazia. | 0 | 1 | 2 | 3 | 4 |
| **2.** Procurar de imediato onde é a casa de banho quando chega a um local desconhecido. | 0 | 1 | 2 | 3 | 4 |
| **3.** Reduzir a ingestão de líquidos. | 0 | 1 | 2 | 3 | 4 |
| **4.** Evitar ir a locais onde desconhece a localização das casas de banho. | 0 | 1 | 2 | 3 | 4 |
| **5.** Ficar mais tempo em casa de forma a evitar situações desconfortáveis. | 0 | 1 | 2 | 3 | 4 |
| **6.** Limitar a atividade física. | 0 | 1 | 2 | 3 | 4 |
| **7.** Antes de se deslocar a um local desconhecido, tentar saber onde se situam as casas de banho. | 0 | 1 | 2 | 3 | 4 |
| **8.** Limitar as saídas sociais. | 0 | 1 | 2 | 3 | 4 |
| **9.** Limitar atividades, como viajar, que requerem estar longe da casa de banho durante um longo período. | 0 | 1 | 2 | 3 | 4 |

|  | Nunca | Raramente | Algumas vezes | Muitas vezes | Todos os dias/Sempre |
| --- | --- | --- | --- | --- | --- |
| **10**. Usar cuecas-fralda. | 0 | 1 | 2 | 3 | 4 |
| **11.** Usar pensos femininos. (higiénicos). | 0 | 1 | 2 | 3 | 4 |
| **12.** Usar outros materiais absorventes (tais como papel higiénico, lenços de papel ou toalhas de papel). | 0 | 1 | 2 | 3 | 4 |
| **13.** Usar cores ou roupas escuras que escondam as manchas. | 0 | 1 | 2 | 3 | 4 |
| **14.** Usar saias e casacos compridos para cobrir as manchas. | 0 | 1 | 2 | 3 | 4 |
| **15.** Usar fraldas de adultos. | 0 | 1 | 2 | 3 | 4 |
| **16.** Usar em excesso perfume ou água de colónia para disfarçar o cheiro da urina. | 0 | 1 | 2 | 3 | 4 |

**Secção Adicional:**

**Urinary Incontinence – Treatment and Management Questionnaire**

**1.** Toma medicação para a Incontinência Urinária?

Sim ___ Não ___ Qual? _________

**2.** Faz atualmente exercícios para fortalecimento do pavimento pélvico (ex., exercícios Kegel), para a Incontinência Urinária?

Sim ___ Não ___

**3.** Já se submeteu a uma cirurgia para recuperação da Incontinência Urinária?

Sim ___ Não ___

**4.** Já alguma vez falou com o seu médico sobre a sua incontinência urinária?

Sim ___ Não ___

**4.1**. Se sim, qual foi a recomendação? __________________________

**Appendix C – Frequency of self-management coping strategies**

| **Items** | **Absolute Frequency**  **(*n*)** | **Relative Frequency (%)** |
| --- | --- | --- |
| ***Defensive Coping*** | | |
| **1. Going to the toilet often, even if you don't feel like it, in order to keep your bladder empty.** | | |
| Never | 511 | 33.22 |
| Rarely | 503 | 32.70 |
| Sometimes | 367 | 23.86 |
| Often | 125 | 8.13 |
| Everyday/Always | 32 | 2.08 |
| **2. Immediately look for the toilet when you arrive in an unfamiliar place.** | | |
| Never | 578 | 37.58 |
| Rarely | 447 | 29.06 |
| Sometimes | 272 | 17.69 |
| Often | 136 | 8.84 |
| Everyday/Always | 104 | 6.76 |
| **3. Reduce your fluid intake.** | | |
| Never | 568 | 36.93 |
| Rarely | 422 | 27.44 |
| Sometimes | 347 | 22.56 |
| Often | 157 | 10.21 |
| Everyday/Always | 45 | 2.93 |
| **4. Avoid going to places where you don't know the location of the toilets.** | | |
| Never | 849 | 55.2 |
| Rarely | 401 | 26.07 |
| Sometimes | 170 | 11.05 |
| Often | 79 | 5.14 |
| Everyday/Always | 40 | 2.60 |
| **5. Staying at home longer to avoid uncomfortable situations.** | | |
| Never | 973 | 63.26 |
| Rarely | 325 | 21.13 |
| Sometimes | 145 | 9.43 |
| Often | 63 | 4.10 |
| Everyday/Always | 32 | 2.08 |
| **6. Limit physical activity.** | | |
| Never | 815 | 52.99 |
| Rarely | 365 | 23.73 |
| Sometimes | 196 | 12.74 |
| Often | 111 | 7.22 |
| Everyday/Always | 51 | 3.32 |
| **7. Before travelling to an unfamiliar place, try to find out where the toilets are located.** | | |
| Never | 918 | 59.69 |
| Rarely | 321 | 20.87 |
| Sometimes | 161 | 10.47 |
| Often | 89 | 5.79 |
| Everyday/Always | 49 | 3.19 |
| **8. Limit social outings.** | | |
| Never | 1058 | 68.79 |
| Rarely | 307 | 19.96 |
| Sometimes | 111 | 7.22 |
| Often | 42 | 2.73 |
| Everyday/Always | 20 | 1.30 |
| **9. Limit activities, such as travelling, that require you to be away from the toilet for a long period of time.** | | |
| Never | 1025 | 66.64 |
| Rarely | 302 | 19.64 |
| Sometimes | 123 | 8.00 |
| Often | 58 | 3.77 |
| Everyday/Always | 30 | 1.95 |
| ***Hiding Coping*** | | |
| **11. Use feminine (hygienic) pads.** | | |
| Never | 339 | 22.04 |
| Rarely | 303 | 19.70 |
| Sometimes | 344 | 22.37 |
| Often | 186 | 12.09 |
| Everyday/Always | 367 | 23.86 |
| **12. Use other absorbent materials (such as toilet paper, tissues or paper towels).** | | |
| Never | 869 | 56.50 |
| Rarely | 345 | 22.43 |
| Sometimes | 226 | 14.69 |
| Often | 64 | 4.16 |
| Everyday/Always | 35 | 2.28 |
| **13. Wear dark colours or clothes that hide the stains.** | | |
| Never | 1068 | 69.44 |
| Rarely | 214 | 13.91 |
| Sometimes | 133 | 8.65 |
| Often | 80 | 5.20 |
| Everyday/Always | 42 | 2.73 |
| **14. Wear long skirts and coats to cover the stains.** | | |
| Never | 1223 | 79.52 |
| Rarely | 152 | 9.88 |
| Sometimes | 89 | 5.79 |
| Often | 50 | 3.25 |
| Everyday/Always | 23 | 1.50 |
